# Evaluation of potential reference genes in the biting midge *Culicoides sonorensis* for real-time quantitative PCR analyses

**DOI:** 10.1038/s41598-023-43750-2

**Published:** 2023-10-04

**Authors:** Cameron Osborne, Anastasia M. W. Cooper, Brandon Hall, Edward Bird, Dana Nayduch, Kristopher Silver

**Affiliations:** 1 Department of Entomology, Kansas State University, 123 W. Waters Hall, 1603 Old Claflin Pl., Manhattan, KS 66506 USA; 2 United States Department of Agriculture-Agricultural Research Service, Manhattan, KS 66502 USA

**Keywords:** Molecular biology, Physiology

## Abstract

Studies examining differentially expressed genes and gene silencing by RNA interference (RNAi) require a set of stably expressed reference genes for accurate normalization. The biting midge *Culicoides sonorensis* is an important vector of livestock pathogens and is often used as a model species for biting midge research. Here, we examine the stable expression of six candidate reference genes in *C. sonorensis*: *actin*, *β-tubulin*, glyceraldehyde 3-phosphate dehydrogenase (*GAPDH*), ribosomal protein subunit (*RPS*) 18, vacuolar ATPase subunit A (*VhaA*), and elongation factor 1-beta (*EF1b*). Gene expression was assessed under seven conditions, including cells treated with double-stranded RNA (dsRNA), 3rd and 4th instar larvae treated with dsRNA, six developmental stages, four adult female body parts or tissue groups, and females injected with bluetongue virus or vesicular stomatitis virus. Stable gene expression was assessed using RefFinder, NormFinder, geNorm, and BestKeeper. The ranked results for each analysis tool under each condition and a comprehensive ranking for each condition are presented. The data show that optimal reference genes vary between conditions and that just two reference genes were necessary for each condition. These findings provide reference genes for use under these conditions in future studies using real-time quantitative PCR to evaluate gene expression in *C. sonorensis*.

## Introduction

Real-time quantitative polymerase chain reaction (RT-qPCR) is an invaluable tool used to examine gene expression, but proper use requires optimization of conditions and identification of appropriate reference genes^1^. Reference genes are those that demonstrate stable expression across variable conditions and are used to calibrate expression relative to a target gene of interest. This type of calibration intends to account for differences in RNA levels among samples and to eliminate non-biological variations due to quality and quantity of template used, the yield of the extraction process, and differences in enzymatic reactions^2,3^. However, the expression of reference gene transcript levels may vary across species, tissues, developmental stages, and in response to biotic and abiotic factors. These variations can subsequently affect measured changes in gene expression and mask real biological significance^4^. Therefore, evaluating and selecting appropriate reference genes for individual study organisms under specific experimental conditions is essential to employ RT-qPCR^3,4^.

*Culicoides* biting midges are economically important livestock pests that have been implicated as vectors of numerous pathogenic viruses and some pathogenic nematodes^5,6^. In the United States, *C. sonorensis* Wirth & Jones (Diptera: Ceratopogonidae) can transmit bluetongue virus (BTV), epizootic hemorrhagic disease virus (EHDV), and vesicular stomatitis virus (VSV), which are concerns primarily for livestock producers^7,8^. *Culicoides sonorensis* is one of the few species to be established in colony, and, as such, has been a model species for laboratory-based studies^9^. Physiological and behavioral changes due to arbovirus infection have been documented previously in *C. sonorensis*^10^. Nayduch et al. found that midges infected with EHDV had 2401 differently expressed unigenes compared to uninfected controls^11^. Many genes associated with tissue structure, sensory processes, vision, and behavior were significantly downregulated in EHDV-infected midges, while genes coding for innate immune responses and olfaction were upregulated. Transcriptome studies like this one are often paired with RT-qPCR to confirm the up- and down-regulation of genes of interest and rely on having a set of stably expressed reference genes for normalization.

Using reference genes in relative RT-qPCR is necessary to fulfill the minimum information for publication of quantitative real-time PCR experiments (MIQE)^12^. Common reference gene candidates in insects include those involved in cytoskeleton structure formation, protein synthesis, and metabolism^3^. In *C. sonorensis*, several ribosomal proteins (*Rp*) were identified in a transcriptome study of adult females but were found to be unsuitable as reference genes in later evaluation due to their variable expression across feeding conditions^13^. This same study found elongation factor 1-beta (*EF1b*) to be a suitable option based on its stable expression across several conditions including unfed or sugar or blood-fed midges, as determined by comparative transcriptomics. The *EF1b* gene was subsequently used as a reference gene in studies of humoral immune responses^14^, EHDV infection^11,15^, and RNA interference (RNAi) studies^16,17^. Other reference genes used in *C. sonorensis* studies include heat shock protein 60 (*HSP60*), cytochrome (*CytB5*), *RpL13*, *RpL21*, *RpS8*, and vacuolar type ATPase (*V-ATPase*) subunits^18,19^. Only one study appears to have used the widely accepted analysis tools NormFinder, geNorm, and BestKeeper to determine suitability of reference genes for normalization of RT-qPCR data in *C. sonorensis*, and this study focused on the vector competence of susceptible and refractory individuals^19^. Since *C. sonorensis* is a model organism for studies involving biting midge behavior, vector competence, population control, and more, additional work is needed to identify stably expressed reference genes suitable for normalization across a broader range of experimental conditions.

The purpose of the present study is to examine six candidate *C. sonorensis* genes for their suitability as reference genes in RT-qPCR-based relative gene expression analyses under laboratory conditions. We determined the relative expression of two structural genes (*actin* and *β-tubulin*), a metabolic enzyme (*GAPDH*), a ribosomal protein subunit (*RPS18*), the previously described *EF1b*, and subunit A of *Vha*. The expression of these genes was examined in cells and larvae treated with double-stranded RNA (dsRNA); in larval, pupal, and adult *C. sonorensis;* in four adult female body parts or tissue groups (head, midgut, remaining gut tract, and the remaining body); and in female midges injected with BTV or VSV. These data identify the most suitable reference genes for examining relative transcript levels using RT-qPCR, will enhance the utility of *C. sonorensis* as a model system, and will aid in the efforts of other researchers to study the midge physiology and the virus-vector interactions that occur in this veterinary pest.

## Results

### Primer efficiencies and Ct values

**Figure 1:**
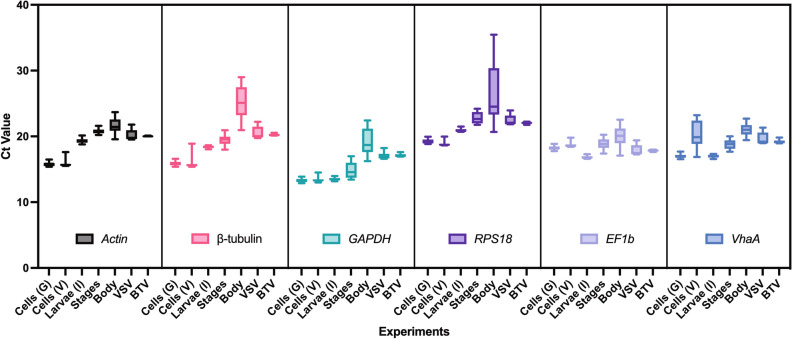
Distribution of Ct values for six *Culicoides sonorensis* candidate reference genes for each of seven experiments. Box and whisker plots showing the 25th to 75th percentiles (box), minimum and maximum Ct values (whiskers), and median Ct value (line). The experiments were cells treated with water or varying concentrations of ds*eGFP* [Cells (G)], cells treated with ds*eGFP* or varying concentrations of ds*VhaA* [Cells (V)], larvae treated with ds*eGFP* or ds*IAP1* [Larvae (I)], midge developmental stages (Stages), midge body parts or tissue groups (Body), and female midges injected with vesicular stomatitis virus (VSV) or bluetongue virus (BTV).

All candidate reference gene RT-qPCR primers had efficiency values between 94.7 and 101.4% (Table 1). Melt curves showed distinct unique peaks for every target analyzed (Supplementary Fig. S1). The spread of Ct values in each experiment and for each target are shown in Fig. 1. All Ct values were below 30, except for *RPS18* in the heads of female midges which were 31.6, 32.9, and 35.2 for the three biological replicates (Supplementary Table S1). These values were included in our analysis. The lowest determined Ct value was 12.9 for *GAPDH*, which belonged to the study of cultured cells treated with varying concentrations of ds*eGFP*.

**Table 1:** Gene target accessions, primers, amplicon size, and primer characteristics of candidate reference genes for *Culicoides sonorensis*.

Target	Accession	Primer sequence (5′- > 3′)	Product size (bp)	Slope	%E	Amp	R^2^
*Actin*	GAWM01012737.1	F: CGATCTGTTGATGCCCGACT	148	3.29	101.39	2.01	0.9982
		R: ATTCGGGCGTGGAAGCTAAC					
*β-tubulin*	GAWM01003055.1	F: CAATCTGGTGCAGGAAACAACT	172	3.46	94.66	1.95	0.9996
		R: GAAGGGTTCCCATGCCTGAA					
*GAPDH*	GAWM01011279.1	F: ACTTGACATGCCGATTGGGT	167	3.34	99.22	1.99	0.9998
		R: GGCCTTGGCGTCAAAGATTG					
*RPS18*	GAWM01018992.1	F: GGCTTAAAACAGAGAAAGGTCTAT	167	3.44	95.28	1.95	0.9989
		R: AATTTGCACAGAATGCAGACTT					
*EF1b*	GAWM01007628.1	F: ATCCGTGAAGAACGTCTCAAA	95	3.37	97.87	1.98	0.9998
		R: CATGGCTTAACTTCGAGGATG					
*VhaA*	GAWM01018658.1	F: GTATGTTGCAAGTGTGGCCTG	86	3.43	95.79	1.96	0.9994
		R: ACGCTGACCAGTTAACAATGGA					

*Abbreviations* bp, Base pair; %E, Primer efficiency; Amp.; Amplification value; *β-tubulin*, Beta-tubulin; *GAPDH*, Glyceraldehyde 3-phosphate dehydrogenase; *RPS18*, Ribosomal protein S18; *VhaA*, Vacuolar-type ATPase subunit A; *EF1b*, Elongation factor 1-beta^13^.

### dsRNA treatments

Comprehensive rankings for the three RNAi experiments are reported in Table 2. We examined whether there would be a difference in the stable gene ranking between cells treated with dsRNA against an exogenous RNAi target (ds*eGFP*) and an endogenous target (ds*VhaA*). The *VhaA* target was selected for these experiments due to considerable cell death (and thus low cellular material) that occurred after dsRNA treatment against inhibitor of apoptosis protein 1 (ds*IAP1*). As expected, *VhaA* was the least stable of the genes tested in this experiment as it was the target for RNAi-based transcript suppression. Overall, the two most stable genes for water- or ds*eGFP*-treated cells were *GAPDH* and *β-tubulin*, and the two for ds*eGFP*- or ds*VhaA*-treated cells were *EF1b* and *GAPDH* (Table 2). The most stable gene in the larval RNAi experiment was *VhaA*. Our findings suggested *β-tubulin* and *EF1b* were the next most stable genes in that order. These gene ranks were inverted in the RefFinder comprehensive ranking (Supplementary Table S3).

**Table 2:** Data output from RefFinder, NormFinder, geNorm, and BestKeeper showing key values used to determine stability, and the rankings (in bold, from 1 highest stability to 6 lowest stability) of each candidate reference gene evaluated in three RNAi experiments in *Culicoides sonorensis* cultured cells and larvae.

Gene	RefFinder		NormFinder		geNorm		BestKeeper					Overall	
	Delta Ct	Rank	Stability value	Rank	Avg. M	Rank	SD [± CP]	r	*p*-value	power [x-fold]	Rank	Geo Mean	Rank
Cells treated with water or ds*eGFP*													
*Actin*	0.106	**6**	0.08	**6**	0.087	**6**	0.24	0.970	0.001	2.01	**4**	5.42	**6**
*β-tubulin*	0.075	**1**	0.04	**1**	0.070	**4**	0.27	0.999	0.001	2.09	**6**	2.21	**2**
*GAPDH*	0.084	**2**	0.05	**3**	0.047	**1**	0.22	0.990	0.001	1.83	**1**	1.57	**1**
*RPS18*	0.087	**5**	0.05	**2**	0.078	**5**	0.27	0.988	0.001	2.05	**5**	3.98	**5**
*EF1b*	0.086	**4**	0.06	**5**	0.047	**2**	0.23	0.982	0.001	1.89	**2**	2.99	**3**
*VhaA*	0.085	**3**	0.05	**4**	0.065	**3**	0.23	0.986	0.001	1.96	**3**	3.22	**4**
Cells treated with ds*eGFP* or ds*VhaA*													
*Actin*	0.682	**3**	0.37	**2**	0.181	**4**	0.32	0.800	0.002	1.87	**4**	3.13	**4**
*β-tubulin*	0.947	**5**	0.61	**5**	0.334	**5**	0.51	0.819	0.001	2.83	**5**	5.00	**5**
*GAPDH*	0.677	**2**	0.41	**4**	0.117	**2**	0.23	0.787	0.002	1.50	**1**	2.00	**2**
*RPS18*	0.687	**4**	0.38	**3**	0.134	**3**	0.24	0.687	0.014	1.41	**2**	2.91	**3**
*EF1b*	0.655	**1**	0.31	**1**	0.117	**1**	0.24	0.857	0.001	1.56	**3**	1.32	**1**
VhaA*	2.31	**6**	1.66	**6**	0.993	**6**	*1.67*	0.461	0.132	3.73	**6**	6.00	**6**
L_3_ and L_4_ larvae treated with ds*eGFP* or ds*IAP1*													
*Actin*	0.254	**5**	0.13	**2**	0.233	**6**	0.27	0.900	0.001	3.02	**6**	5.48	**6**
*β-tubulin*	0.217	**3**	0.09	**3**	0.172	**2**	0.15	0.777	0.003	1.62	**1**	2.06	**2**
*GAPDH*	0.266	**6**	0.13	**6**	0.215	**5**	0.19	0.679	0.015	1.71	**4**	5.18	**5**
*RPS18*	0.242	**4**	0.11	**4**	0.190	**4**	0.21	0.785	0.002	1.99	**5**	4.23	**4**
*EF1b*	0.201	**2**	0.07	**2**	0.180	**3**	0.17	0.862	0.001	1.87	**2**	2.21	**3**
*VhaA*	0.193	**1**	0.07	**1**	0.172	**1**	0.19	0.897	0.001	2.00	**3**	1.32	**1**

*These experiments contained samples treated with ds*VhaA*. The top two candidate genes share the same Avg. M values in the geNorm output. Italics values are above the 1.0 threshold for recommended use. *Abbreviations* M, Stability measure; SD, Standard deviation; CP, Crossing point (i.e., Ct value), Geo Mean, Geometric mean; *β-tubulin*, Beta-tubulin; *GAPDH*, Glyceraldehyde 3-phosphate dehydrogenase; *RPS18*, Ribosomal protein S18; *VhaA*, Vacuolar-type ATPase subunit A; *EF1b*, Elongation factor 1-beta.

### Developmental stages, body parts, and tissue groups

Comprehensive rankings for the life history and body parts or tissue groups studies are reported in Table 3. The consensus ranking from this study and RefFinder found *actin* and *VhaA* to be the most stable candidates when comparing *C. sonorensis* developmental stages (Supplementary Table S3). *GAPDH* was the least stable, and the BestKeeper reported standard deviation (SD) value was above the acceptable threshold (Table 3). The body part and tissue group study found four of the six candidate genes were unacceptable when using the BestKeeper SD cutoff. Only *actin* and *VhaA* were deemed acceptable according to the BestKeeper SD cutoff, but the overall ranking based on all analyses found *VhaA* was fourth. In contrast, even though the Bestkeeper SD was greater than one for *EF1b*, *EF1b* and *actin* were ranked as first and second most stable in the overall rankings, respectively. The tissue study also found *RPS18* to be the worst candidate, and the Ct values for this gene were the most variable of those examined (Fig. 1).

**Table 3:** Data output from RefFinder, NormFinder, geNorm, and BestKeeper showing key values used to determine stability, and the rankings of each candidate reference gene (in bold) evaluated in six developmental stages and four female body parts or tissue groups of *Culicoides sonorensis.*

Gene	RefFinder		NormFinder		geNorm		BestKeeper					Overall	
	Delta Ct	Rank	Stability Value	Rank	Avg. M	Rank	SD [± CP]	r	*p*-value	power [x-fold]	Rank	Geo Mean	Rank
Developmental stages													
*Actin*	0.765	**1**	0.12	**1**	0.487	**3**	0.31	0.887	0.001	1.96	**1**	1.32	**1**
*β-tubulin*	1.071	**5**	0.71	**5**	0.652	**4**	0.67	0.441	0.068	2.05	**4**	4.47	**5**
*GAPDH*	1.681	**6**	1.41	**6**	1.061	**6**	1.06	− 0.018	0.945	0.96	**6**	6.00	**6**
*RPS18*	0.99	**3**	0.66	**4**	0.331	**2**	0.77	0.457	0.056	2.11	**5**	3.31	**3**
*EF1B*	1.001	**4**	0.62	**3**	0.750	**5**	0.63	0.836	0.001	3.76	**3**	3.66	**4**
*VhaA*	0.855	**2**	0.5	**2**	0.331	**1**	0.59	0.624	0.006	2.29	**2**	1.68	**2**
Female body parts and tissue groups													
*Actin*	1.487	**3**	0.9	**3**	0.525	**1**	0.86	0.960	0.001	1.48	**2**	2.06	**2**
*β-tubulin*	1.813	**5**	0.98	**4**	1.118	**5**	1.86	0.932	0.001	2.27	**5**	4.73	**5**
*GAPDH*	1.421	**2**	0.37	**1**	0.801	**4**	1.66	0.984	0.001	2.01	**4**	2.38	**3**
*RPS18*	3.345	**6**	2.21	**6**	1.861	**6**	3.63	0.963	0.001	4.73	**6**	6.00	**6**
*EF1B*	1.396	**1**	0.76	**2**	0.525	**2**	1.15	0.957	0.001	1.66	**3**	1.86	**1**
*VhaA*	1.702	**4**	1.3	**5**	0.623	**3**	0.70	0.880	0.001	1.33	**1**	2.78	**4**

The top two candidate genes share the same Avg. M values in the geNorm output. Underlined values are above the 1.0 threshold for recommended use. *Abbreviations* M, Stability measure; SD, Standard deviation; CP, Crossing point (i.e., Ct value), Geo Mean, Geometric mean; *β-tubulin*, Beta-tubulin; *GAPDH*, Glyceraldehyde 3-phosphate dehydrogenase; *RPS18*, Ribosomal protein S18; *VhaA*, Vacuolar-type ATPase subunit A; *EF1b*, elongation factor 1-beta.

### Virus infections

Comprehensive rankings for the two virus injection studies are reported in Table 4. The top three candidate reference genes for both virus experiments tested were *EF1b*, *actin*, and *RPS18*. Our analyses found *EF1b* to be the most stable in both experiments. *RPS18* was the second most stable for VSV-infected midges, and *actin* was the second most stable for BTV-infected midges. Injecting virus into midges was chosen over the more natural route of feeding midges on virus-spiked blood. A previous study found significant variability in virus infection rates when *C. sonorensis* fed on virus-spiked blood in an artificial system^20^. Virus injection is often preferred to ensure consistent, high levels of infection in *C. sonorensis*; however, it induces higher levels of trauma. We determined the infection rates were more important to the study’s outcome and opted for this method.

**Table 4:** Data output from RefFinder, NormFinder, geNorm, and BestKeeper showing key values used to determine stability, and the rankings of each candidate reference gene (in bold) evaluated in *Culicoides sonorensis* females injected with vesicular stomatitis virus (VSV) and media controls, and bluetongue virus (BTV) and media controls.

Gene	RefFinder		NormFinder		geNorm		BestKeeper					Overall	
	Delta Ct	Rank	Stability value	Rank	Avg. M	Rank	SD [± CP]	r	*p*-value	power [x-fold]	Rank	Geo Mean	Rank
VSV-injected females													
* Actin*	0.204	**3**	0.13	**3**	0.085	**1**	0.69	0.992	0.001	2.07	**4**	2.45	**3**
* β-tubulin*	0.234	**5**	0.16	**5**	0.143	**5**	0.80	0.995	0.001	2.19	**6**	5.23	**6**
* GAPDH*	0.458	**6**	0.36	**6**	0.248	**6**	0.37	0.927	0.008	1.56	**1**	3.83	**4**
* RPS18*	0.188	**2**	0.06	**2**	0.085	**2**	0.66	0.997	0.001	1.95	**2**	2.00	**2**
* EF1b*	0.185	**1**	0.03	**1**	0.106	**3**	0.68	0.999	0.001	2.00	**3**	1.73	**1**
* VhaA*	0.221	**4**	0.16	**4**	0.133	**4**	0.79	0.995	0.001	2.18	**5**	4.23	**5**
BTV-injected females													
* Actin*	0.184	**2**	0.02	**2**	0.110	**2**	0.05	0.733	0.098	1.27	**1**	1.68	**2**
* β-tubulin*	0.206	**4**	0.07	**4**	0.113	**3**	0.12	0.591	0.218	1.63	**4**	3.72	**4**
* GAPDH*	0.277	**5**	0.12	**5**	0.177	**5**	0.19	0.641	0.170	2.48	**5**	5.00	**5**
* RPS18*	0.199	**3**	0.06	**3**	0.136	**4**	0.10	0.725	0.102	1.83	**2**	2.91	**3**
* EF1b*	0.167	**1**	0.02	**1**	0.110	**1**	0.12	0.954	0.003	2.10	**3**	1.32	**1**
* VhaA*	0.325	**6**	0.15	**6**	0.226	**6**	0.21	0.596	0.213	2.73	**6**	6.00	**6**

The top two candidate genes share the same Avg. M values in the geNorm output. *Abbreviations* M, Stability measure; SD, Standard deviation; CP, Crossing point (i.e., Ct value), Geo Mean, Geometric mean; *β-tubulin*, Beta-tubulin; *GAPDH*, Glyceraldehyde 3-phosphate dehydrogenase; *RPS18*, Ribosomal protein S18; *VhaA*, Vacuolar-type ATPase subunit A; *EF1b,* Elongation factor 1-beta.

### Optimal number of reference genes

The geNorm package reports pairwise variation scores (V scores) to inform the number of genes to use for optimal normalization. Scores above the suggested 0.15 cutoff suggest that another gene needs to be added in experimental analysis^21^. None of the V scores in this study reached the 0.15 cutoff. The highest V score among all the experiments was 0.02785, which is well below the cutoff value (Supplementary Table S4). These results suggest that just two reference genes are sufficient to yield accurate results for all the conditions examined here.

## Discussion

This study provides a comprehensive examination of candidate reference genes for analysis of gene expression in *C. sonorensis*. We included five different functional classes of genes and examined their stability in RNAi studies, midge developmental stages, female midge body parts and tissue groups, and with and without virus infections. We excluded teneral, sugar-fed, and blood-fed females from this study as these conditions were examined in a previous transcriptome study^13^. It is widely accepted that at least two reference genes are necessary for adequate normalization when performing relative quantification of transcript levels^12^. Our results suggest that including only two reference genes is suitable for the conditions we tested based on pairwise V scores. Future studies can use the most stably expressed genes described here when normalizing gene expression across similar treatments or evaluate new reference genes using the analysis tools described here.

Our study evaluated two genes that have previously been studied as candidate reference genes. First, *EF1b* was used as a reference gene for viral infection studies, and our findings support this as the most stable gene for these analyses, albeit with a similar but not identical virus^11,15^. Another study used *EF1b* as a reference gene for tissue-specific expression of antimicrobial peptide genes^14^. Second, a V-ATPase subunit was examined for stability among four other candidate reference genes^19^. The authors found V-ATPase unsuitable for inclusion in their study as it ranked poorly against the others when examined with BestKeeper, geNorm, and NormFinder. Of note, that study appears to have used the gene coding subunit C, whereas our study used the gene for the A subunit. *VhaA* was in the top two most stable reference genes in two of our experiments (developmental stages and larvae treated with ds*IAP1*).

Female midge body parts and tissue groups were the most inconsistent samples tested for stable gene expression. The distribution of Ct values from these samples was broad for *β-tubulin*, *GAPDH*, *RPS18*, and *EF1b* (Fig. 1). Unsurprisingly, these four genes were flagged as unacceptable in the BestKeeper software (Table 3). The two remaining genes, *VhaA* and *actin*, were below the suggested cutoff threshold and are, therefore, the only two candidate genes acceptable for female midge tissues studies using the BestKeeper criteria. These insects are incredibly small, just 2 mm long, and dissecting them for individual tissues can be challenging. While every effort was made to preserve the integrity of the samples, it is possible that contaminants from other tissues were included in pooled tissue samples. Evaluating additional reference genes and examining more tissue types is warranted, given our findings.

Insect manipulation during experimentation likely elicited physiological responses in the insects. For example, larvae were moved from a rearing environment containing food, bacteria, and algae into water during the larval treatment experiments. This is a common approach for bioassays where larvae are treated with dsRNA in simple media. No larvae died during the experiments described in this study, but stress from the environmental change could have altered gene expression if compared to un-stressed larvae. However, changes in expression of transcript levels between *Culicoides* larvae undergoing standard experimental protocols versus no experimental treatment was not the goal of our investigations. Instead, we assessed the effect of treatments used in our experimental protocols on the stability and suitability of housekeeping genes for RT-qPCR.

The RefFinder website provides a simple user interface for examining stable expression among candidate reference genes using only raw Ct values. RefFinder generates a report for the other analysis tools (NormFinder, BestKeeper, geNorm) and gives a stability rank for each gene submitted. Additionally, the website incorporates a comparative delta Ct analysis, yielding a fourth stability ranking. The geometric mean of each rank is used to create a comprehensive stability rank for the submitted genes. However, the tool’s input field does not use transformed data (e.g., as in NormFinder), nor does it have an input for primer efficiencies (e.g., as in BestKeeper). We examined how the RefFinder ranks compared to those generated in our analyses and reported the findings in Supplementary Table S3. Our dsRNA-treated cell experiments, developmental stage analysis, body part and tissue group analysis, and BTV-injected females experiment had identical comprehensive rankings. The remaining two experiments, dsRNA-treated larvae and VSV-injected females, had two inverted and one inverted gene ranks, respectively. The gene rank disagreement in VSV-injected females did not alter which genes are recommended for normalization (i.e., *RPS18* and *EF1b*). Taken together, these findings suggest that the RefFinder website tool alone may be adequate for preliminary stable reference gene stability analyses.

Around the world, *Culicoides* are a significant threat to livestock production and, in some instances, human health. Ongoing and future research will rely on the use of reference genes in gene expression studies to examine vector competency, behavior, RNAi-based gene suppression, and other studies of midge biology and ecology. We have found only a handful of studies that have evaluated reference genes for differential gene analysis in *C. sonorensis.* This species will continue to play a prominent role as a model for numerous studies given that it is one of the few midge taxa that can be colony reared. Each of the six candidate genes examined in this study appear at least once as the most stable gene among all of our experimental conditions. This finding indicates that there is no singular reference gene, or set of genes, which could be considered universal for all *C. sonorensis* experiments. In fact, no universal reference genes demonstrating stable expression across species, developmental stages, tissues, and experimental conditions have been identified in any species to date, reinforcing the need to validate reference gene expression stability before every RT-qPCR experiment^3,4^.

## Methods

### Insect cells and developmental stages

*Culicoides sonorensis* cells (W8A) are of embryonic origin and were maintained in Schneider’s Insect Media (Sigma-Aldrich, St. Louis, MO, USA) (24.5 g/L) supplemented with 0.4 g/L sodium bicarbonate, 0.0585 g/L l-glutamine, 0.006 g/L reduced glutathione, 0.03 g/L l-asparagine, 0.6 g/L calcium chloride, 18 μL of 10 mg/L bovine insulin and 15% fetal bovine serum at 28 °C^22^. The AK line of *C. sonorensis* was used for all developmental stages, body part and tissue group collection, and virus injection studies^9^. Insects were kept at 25 °C and 60–80% relative humidity with a 14:10 light:dark cycle during the duration of the experiments unless otherwise noted. Both cultured cells and insects were sourced from the USDA-ARS facility in Manhattan, Kansas where they are actively maintained.

### Experimental treatments

#### dsRNA synthesis

Pooled cDNA (described below) was used to synthesize dsRNA. Briefly, primers containing a T7 promoter sequence were used to amplify DNA template from *C. sonorensis* cDNA (ds*IAP1* and ds*VhaA*) or from a plasmid containing *eGFP* (Supplementary Table S2). Reaction mixtures were as follows: 25 μl of 2X PCR MasterMix with Dye (abm), 2.5 μl each of forward and reverse primer (10 μM stock), 2 μl of cDNA template, and 18 μl of nuclease-free water. Thermal cycling conditions were 94 °C for 3 min, 35 cycles of denaturation at 94 °C for 30 s, annealing at 50 °C for 30 s, and extension at 72 °C for 1 min, and a final extension step of 72 °C for 5 min. The PCR product was run on and extracted from a 1.5% agarose gel and purified with a gel extraction kit (QIAGEN, Hilden, Germany). Double-stranded RNA was synthesized from 1 μg of cDNA template using a HiScribe T7 High Yield RNA synthesis kit (NEB, Ipswich, MA) according to manufacturer’s recommendations. The dsRNA product was purified by sodium acetate–ethanol precipitation overnight at − 20 °C, pelleted by centrifugation at 17,000 × g for 10 min, washed twice with 70% ethanol, and reconstituted in nuclease-free water. Then, dsRNA was quantified by spectroscopy (Implen, Westlake Village, CA) and evaluated for specificity by gel electrophoresis.

#### Cells treated with dsRNA

Cultured *C. sonorensis* cells were seeded into 12-well culture plates in 1 mL of modified Schneider's media and grown to ~ 75% confluence. The first experiment examined cell responses to non-target dsRNA by treating wells with 1 μg (i.e., 1 μg/mL), 100 ng, or 10 ng of ds*eGFP*, or nuclease-free water as a control. Each treatment and control were replicated in triplicate. Cells were harvested 48 h post-treatment (hpt) in 400 μl of TRIzol reagent (Invitrogen, Waltham, MA) and processed for RNA extraction as below. By this time point cells had reached ~ 90% confluency. A second experiment examined cell response to an endogenous target by using ds*VhaA*. Wells were treated with 10 μg, 1 μg, or 100 ng of ds*VhaA*, or 1 μg of ds*eGFP* as a control. Cells were harvested at 48 hpt as above.

#### Larvae treated with dsRNA

Instar 3 (L_3_) or 4 (L_4_) larvae were aliquoted into wells of a 12-well cell culture plate (n = 20 each) and the rearing water was aspirated and replaced with 1 mL nuclease-free water. The experimental layout consisted of 3 biological replicates (pools of 20 larvae) for each instar and each condition (control or treatment). Each well was treated with 10 μg (i.e., 10 μg/mL) of dsRNA targeting either *IAP1* or *eGFP*. Larvae were maintained in a growth chamber for 72 h before being harvested in 400 μl TRIzol reagent. All larvae survived the treatments, and the total pool was used for each biological replicate. Larvae were homogenized with a tissue pestle, centrifuged at 16,000 × g for 5 min to pellet the bulk insect tissue, and then the liquid portion was moved to a new, clean tube for extraction of total RNA.

#### Developmental stage, body part, or tissue group collection

L_3_ and L_4_ larvae, sex-sorted male and female pupae^23^, and adult male and female midges (sugar-fed) were collected directly into 400 μl TRIzol reagent. Three pools of each stage were collected and consisted of 20 individuals per pool (n = 20). Pooled insects were homogenized with two 2.4 mm stainless steel beads in a Bead Mill Homogenizer (Omni International, Kennesaw GA). Homogenized pools were centrifuged as above to pellet bulk tissue and the liquid portion was moved to a new tube for total RNA extraction. Adult female midges (n = 50) were individually dissected in drops of nuclease-water to yield four body parts or tissue groups: 1) heads with salivary glands attached, 2) midguts separated from 3) remaining gut and associated organs (foregut, hindgut, and Malpighian tubules), and 4) the remaining body of the midge. Three pools of each tissue were collected in 400 μl of TRIzol, homogenized with a pestle, and processed as above.

#### Virus injected midges

Adult *C. sonorensis* midges were obtained from the AK colony maintained by USDA-ARS in Manhattan, KS. Mated female midges (3–4 days post-eclosion) were anesthetized with CO_2_ and injected with 60 nl of either VSV-NJ (2.52 X 10^8^ PFU/ml) grown in porcine epithelial cells (AG08113; Coriell Institute, Camden, NJ) or BTV-17 (6.63 X 10^5^ PFU/ml) grown in baby hamster kidney cells (BHK, American Type Culture Collection, Manassas, VA). Injections were delivered intrathoracically into the soft cuticle between the wing base and second pleural sclerite using a hand-held Nanoject II (Drummond Scientific Company, Broomall, PA). After treatment, midges were offered 10% sucrose ad libitum and were maintained in environmental chambers at 25 ± 1 °C and 75 ± 5% relative humidity with a 13:11 light:dark cycle. Six days post-injection (dpi), three pools of each treatment were collected and consisted of 20 individuals per pool (n = 20). Midge pools were homogenized with two stainless steel beads in 400 μl TRIzol as above.

#### RNA extraction and cDNA synthesis

Total RNA was isolated using TRIzol according to the manufacturer's instructions (Fisher Scientific, Pittsburgh, PA). Total RNA was reconstituted in 50 μl of nuclease-free water. Final RNA concentrations were measured by spectroscopy, and cDNA was synthesized using 1 μg of RNA with an OneScript® Plus cDNA Synthesis Kit (abm, Richmond, BC, Canada). Residual genomic DNA was removed by incubation with DNaseI (Thermo Fisher Scientific, Waltham, MA) for 30 min at 37 °C followed by enzyme inactivation with EDTA at 65 °C for 10 min. The resulting cDNA was diluted 1:3 with nuclease-free water to create the template for RT-qPCR.

#### Genes selection and RT-qPCR primers

Candidate reference genes were selected based on published literature on mosquitoes, other insects, and other studies using *C. sonorensis*^3,13,24^. These candidate genes were from multiple functional classes, including structural proteins, enzymes involved in metabolism, subunits of the ribosome and vacuolar ATPase, and a protein elongation factor. Transcript sequences were obtained from a transcriptome shotgun assembly (Accession: PRJNA238338) which is hosted on the National Center for Biotechnology Information (NCBI) database, and primers were designed using the Primer-BLAST web tool (https://www.ncbi.nlm.nih.gov/tools/primer-blast/). Optimal primers were designed to have 18–22 nucleotide bases, an amplicon size of 80–200 bp, 60 °C melt temperature, 50–60% GC content, and specificity to *C. sonorensis*. The RT-qPCR primers, amplicon size, and transcript accessions are reported in Table 1.

#### Real-time quantitative PCR

Primers were validated using pooled cDNA from multiple midge developmental stages. The cDNA was initially diluted 1:20 with nuclease-free water and then used to create a 1:10 dilution series for a standard curve. Melt curve analyses were performed to ensure primer specificity. All quantifications were performed on a QuantStudio 7 Pro (Thermo Fisher Scientific). Reaction mixtures were as follows: 10 μl of BlasTaq™ 2X RT-qPCR MasterMix (abm) with 1 μl of ROX reference dye per 1.25 mL MasterMix, 0.5 μl of each primer (10 μM stocks), 1 μl of cDNA template, and 8 μl of molecular grade water. Thermal cycling conditions consisted of a 3 min hold at 95 °C, 40 cycles of denaturing at 95 °C for 15 s and annealing and extension at 60 °C for 1 min. All sample reactions were performed in duplicate (technical replicates).

### Data analysis

Technical replicate Ct values were averaged for analysis and these values are reported from each experimental condition in Supplementary Table S1. No-template controls were all negative or had Ct values above 36 which were considered negligible. Raw Ct values were used for RefFinder^25^, BestKeeper^26^, and geNorm^21^. Data were transformed using the equation RQ = E^−(Cq min−Cq sample)^ for NormFinder^27^, where relative quantity (RQ) is determined using the primer efficiency (E) and the difference between the minimum Ct value in the gene group and each Ct sample. Some of the spreadsheet-based tools could not be used on some computers (e.g., NormFinder), and one hosted on a website had discontinued support (e.g., GeNorm). Both of these tools have been adapted for use in R and we successfully ran scripts provided by developers (geNorm, https://search.r-project.org/CRAN/refmans/ctrlGene/html/00Index.html; NormFinder, https://moma.dk/normfinder-software). The NormFinder package for R also offers flexibility in using raw Ct or linearly transformed values. Both geNorm and NormFinder analyses were performed in R Studio (version 2022.07.2 + 576) for this study.

RefFinder uses a delta Ct approach to rank genes with the lowest variability across conditions as the most ideal. The NormFinder stability value is derived from inter- and intra-sample variation, which are combined to measure systemic error, and the lower the value the better the candidate gene is ranked. The geNorm analysis yields an average M value which is derived from the expression ratios between any two candidate reference genes. Those pairs with the lowest expression ratios are ranked higher as they’re more like one-another. The top pair of candidate genes are reported with the same average M value. The most stable genes from BestKeeper were ranked first by the lowest standard deviation and then by the lowest coefficient of variance. Candidate reference genes with a standard deviation greater than one are not recommended for use^26^. Stability rankings from each analysis were assigned to each gene under each condition tested, and the geometric mean of these values was used to create a cumulative ranking. This is the same approach that’s available in the RefFinder web application. The gene stability ranks determined in this study were compared to the ranks generated by RefFinder to evaluate their consensus.

### Supplementary Information

Supplementary Information.

## Data Availability

All data generated and analyzed for this study are included in this published article and its Supplementary Information files. All programs used to analyze the data are publicly available.
