# Supplementary material for: Evaluation of potential reference genes in the biting midge Culicoides sonorensis for real-time quantitative PCR analyses
Source: Sci Rep. 2023 Oct 4;13:16729. doi: 10.1038/s41598-023-43750-2 (PMC10550929; doi:10.1038/s41598-023-43750-2)
Supplement: Supplementary file 1 — Supplementary Information. [file 41598_2023_43750_MOESM1_ESM.pdf]

Supplementary Information

Evaluation of potential reference genes in the biting midge *Culicoides sonorensis* for real-time quantitative PCR analyses

Cameron Osborne[1], Anastasia M. W. Cooper[1], Brandon Hall[1], Edward Bird[1], Dana Nayduch[2], and Kristopher Silver[1]*

[1]Department of Entomology, Kansas State University, Manhattan, KS 66506, USA

[2]United States Department of Agriculture-Agricultural Research Service, Manhattan, KS 66502, USA

*Corresponding author: Kristopher Silver, 123 W. Waters Hall, 1603 Old Claflin Pl., Manhattan, KS 66506, USA. 785-532-4752. ksilver@ksu.edu.

Supplementary Table S1

Reported Ct values of six candidate reference genes for seven experiments in this study. Three biological replicates (each the average of two technical replicates) are reported for each experimental condition. Reference genes are *actin*, beta-tubulin (*β-tubulin)*, glyceraldehyde 3-phosphate dehydrogenase (*GAPDH)*, ribosomal protein S18 (*RPS18)*, vacuolar-type ATPase subunit A (*VhaA), and* elongation factor 1-beta (*EF1b)*.

	Actin	*β-tubulin*	*GAPDH*	*RPS18*	*EF1b*	*VhaA*
**Cells treated with water or ds*eGFP***						
Water	15.78	15.79	13.16	19.18	18.16	16.92
	16.48	16.59	13.88	19.96	18.85	17.69
	15.87	15.74	13.20	19.20	18.10	16.85
1 µg/mL ds*eGFP*	16.29	16.26	13.59	19.65	18.48	17.39
	15.95	16.15	13.57	19.56	18.50	17.24
	15.91	16.05	13.43	19.43	18.42	17.01
100 ng/mL ds*eGFP*	15.88	15.91	13.32	19.38	18.23	16.99
	15.76	15.78	13.19	19.21	18.12	16.86
	15.47	15.38	12.88	18.89	17.77	16.53
10 ng/mL ds*eGFP*	15.47	15.58	13.07	18.91	18.05	16.74
	15.59	15.69	13.24	19.01	18.16	16.91
	15.39	15.44	13.00	18.84	17.84	16.69
**Cells treated with ds*eGFP* or ds*VhaA***						
1 µg/mL ds*eGFP*	15.99	15.93	13.45	19.21	18.88	17.18
	17.62	18.90	14.51	19.97	19.81	19.57
	15.69	15.49	13.30	18.88	18.50	16.87
10 µg/mL ds*VhaA*	15.76	15.69	13.52	18.82	18.82	23.01
	15.73	15.63	13.26	18.69	18.65	23.23
	15.52	15.38	13.25	18.63	18.57	23.22
1 µg/mL ds*VhaA*	15.63	15.43	13.22	18.73	18.48	20.18
	15.57	15.60	13.06	18.73	18.41	20.41

	15.58	15.44	12.99	18.72	18.46	20.56
100 ng/mL ds*VhaA*	15.82	15.84	13.49	18.85	18.63	19.20
	15.60	15.48	13.33	18.63	18.44	18.81
	15.64	15.65	13.38	18.71	18.51	18.97
**L_3 and L_4 larvae treated with ds*eGFP* or ds*IAP1***						
L_3: 10 µg/mL ds*eGFP*	19.62	18.61	13.77	21.44	17.29	17.20
	19.62	18.66	13.59	21.50	17.12	17.21
	18.95	18.21	13.21	20.77	16.68	16.78
L_4: 10 µg/mL ds*eGFP*	18.79	18.04	13.18	20.84	16.64	16.55
	20.14	18.50	13.46	21.14	17.02	17.34
	19.40	18.45	13.49	20.96	16.90	16.99
L_3: 10 µg/mL ds*IAP1*	19.25	18.38	13.31	20.80	16.70	16.75
	19.03	18.21	13.30	20.83	16.67	16.87
	19.34	18.28	13.97	20.70	16.83	16.86
L_4: 10 µg/mL ds*IAP1*	19.41	18.46	13.68	21.03	16.60	17.00
	19.06	18.64	13.44	20.72	16.64	16.92
	19.40	18.35	13.64	20.79	16.84	17.26
**Developmental Stages**						
L_3 Larvae	20.72	19.72	13.73	23.66	18.24	19.12
	20.76	18.99	14.01	23.64	18.28	18.93
	21.00	19.78	14.25	23.81	18.45	20.00
L_4 Larvae	21.12	19.73	13.56	24.21	18.94	19.65
	20.89	19.60	13.44	23.98	18.81	19.61
	20.96	19.36	13.55	23.83	18.78	19.64
Male Pupae	20.52	18.58	16.11	21.99	19.03	18.35
	20.93	19.18	16.99	22.44	19.45	18.75
	20.84	18.91	15.97	22.36	19.80	18.62
Female Pupae	20.38	17.99	16.53	21.84	18.63	18.14
	20.51	18.79	16.62	22.19	18.97	18.16
	20.21	18.05	15.89	21.76	18.91	18.00
Males	21.60	20.95	14.97	23.52	20.26	19.32

	21.18	20.68	13.83	22.85	19.85	18.92
	21.28	20.80	13.64	22.97	20.16	19.04
Females	20.36	19.83	15.36	21.86	17.39	17.99
	20.22	19.49	14.85	21.93	17.72	17.67
	20.55	20.29	14.30	22.20	17.82	18.00
**Female Body Parts and Tissue Groups**						
Midgut	21.63	24.82	19.77	26.06	20.50	21.66
	21.02	23.77	19.04	25.08	20.05	21.04
	21.70	25.25	20.00	26.54	20.69	21.73
Remaining Gut	19.56	20.97	16.24	20.69	17.08	19.70
	20.54	21.37	17.30	21.77	18.92	19.43
	21.83	23.03	18.34	23.32	20.11	20.99
Remaining Body	20.82	25.51	17.59	23.95	19.12	20.15
	21.25	25.01	17.65	23.33	18.77	20.96
	21.06	25.19	17.71	23.43	19.30	20.68
Head	23.68	29.00	22.43	31.65	22.54	22.71
	22.83	28.27	21.58	32.87	21.36	21.36
	22.85	28.13	21.80	35.46	21.79	21.80
**VSV-Injected Females**						
Media Injected	19.78	19.75	16.59	21.91	17.25	19.04
	20.67	21.22	17.08	22.89	18.41	20.34
	21.78	22.22	18.22	23.95	19.39	21.32
VSV Injected	19.77	20.05	17.06	22.01	17.54	19.11
	19.63	19.97	16.68	21.99	17.31	18.97
	19.52	19.89	17.01	21.84	17.38	19.05
**BTV-Injected Females**						
Media Injected	20.01	20.19	17.03	22.06	17.70	18.97
	19.97	20.13	16.94	21.75	17.63	18.95
	20.03	20.21	17.01	22.02	17.82	19.17
BTV Injected	20.10	20.54	17.19	22.07	17.99	19.16
	20.12	20.32	17.62	22.05	17.94	19.23

	19.98	20.13	16.92	22.24	17.92	19.82

Supplementary Figure S1. Melt curve analyses of six *Culicoides sonorensis* candidate reference genes. Analysis was performed on a QuantStudio 7 Pro (Thermo Fisher Scientific). Outputs are from the Design & Analysis Software (Release Version: 2.6.0, Thermo Fisher Scientific). The figure shows the melt curves for *EF1b* (a), *β-tubulin* (b), *VhaA* (c), *actin* (d), *RPS18* (e), and *GAPDH* (f).

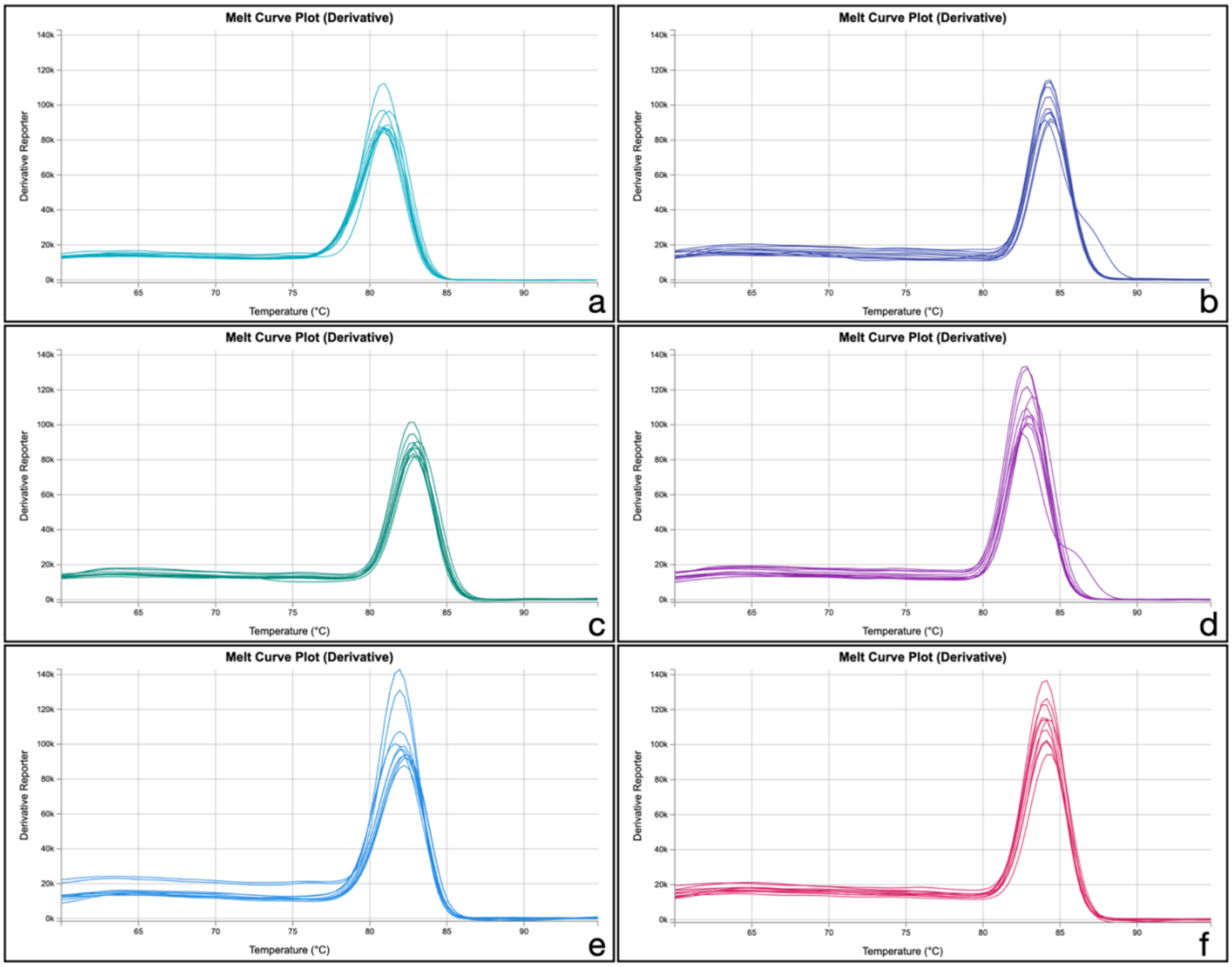

Supplementary Table S2. Primers for dsRNA synthesis for *C. sonorensis* experiments.

Target	Accession/Source	Primer Sequence (5'->3')	Product Size (bp)
ds*IAP1*[1]	GAWM01009039.1	F: [T7]-AGTTGAAGAACACTTGAGATGG	344
		R: [T7]-GCCAATCTTCATACGACACC	
ds*VhaA*	GAWM01018658.1	F: [T7]-CAACTGCAATACCAGGAGCG	409
		R: [T7]-CGTGCACCTAAATATGCGGG	
ds*eGFP*[2]	Ac5-STABLE1-neo (Plasmid #32425)	F: [T7]-TGACCACCCTGACCTAC	304
		R: [T7]-TTGATGCCGTTCTTCTGC	

Abbreviations: bp, base pair; ds, double-stranded; *IAP1*, inhibitor of apoptosis 1; *VhaA*, vacuolar-type ATPase subunit A; *eGFP*, enhanced green fluorescent protein; [T7], T7 Promoter Sequence (TAATACGACTCACTATAGGG). Primer references are superscripted. Ac5-STABLE1-neo was a gift from Rosa Barrio & James Sutherland (Addgene plasmid # 32425 ; http://n2t.net/addgene:32425 ; RRID:Addgene_32425).

Supplementary Table S3. Rankings from 1 (best) to 6 (worst) of six candidate *C. sonorensis* reference genes under seven experimental conditions as determined by four analysis tools (RefFinder, BestKeeper, NormFinder, and geNorm). Comprehensive rankings are reported from RefFinder and this study's analyses.

		Ranking					
Experiment	Algorithm	1	2	3	4	5	6
Cells (ds*eGFP*)	RF[A]: Delta Ct	*β-tubulin*	*GAPDH*	*VhaA*	*EF1b*	*RPS18*	*Actin*
	RF: BestKeeper	*GAPDH*	*EF1b*	*VhaA*	*Actin*	*β-tubulin*	*RPS18*
	RF: Normfinder	*β-tubulin*	*VhaA*	*GAPDH*	*RPS18*	*EF1b*	*Actin*
	RF: geNorm	*GAPDH*	*EF1b*	*VhaA*	*β-tubulin*	*RPS18*	*Actin*
	**RF: Comprehensive**	***GAPDH***	***β-tubulin***	***EF1b***	***VhaA***	***RPS18***	***Actin***
	BestKeeper	*GAPDH*	*EF1b*	*VhaA*	*Actin*	*RPS18*	*β-tubulin*
	NormFinder	*β-tubulin*	*RPS18*	*GAPDH*	*VhaA*	*EF1b*	*Actin*
	geNorm	*GAPDH*	*EF1b*	*VhaA*	*β-tubulin*	*RPS18*	*Actin*
	**Comprehensive**	***GAPDH***	***β-tubulin***	***EF1b***	***VhaA***	***RPS18***	***Actin***
Cells (ds*VhaA*)	RF: Delta Ct	*EF1b*	*GAPDH*	*Actin*	*RPS18*	*β-tubulin*	*VhaA*
	RF: BestKeeper	*GAPDH*	*RPS18*	*EF1b*	*Actin*	*β-tubulin*	*VhaA*
	RF: Normfinder	*EF1b*	*GAPDH*	*Actin*	*RPS18*	*β-tubulin*	*VhaA*
	RF: geNorm	*GAPDH*	*EF1b*	*RPS18*	*Actin*	*β-tubulin*	*VhaA*
	**RF: Comprehensive**	***EF1b***	***GAPDH***	***RPS18***	***Actin***	***β-tubulin***	***VhaA***
	BestKeeper	*GAPDH*	*RPS18*	*EF1b*	*Actin*	*β-tubulin*	*VhaA*
	NormFinder	*EF1b*	*Actin*	*RPS18*	*GAPDH*	*β-tubulin*	*VhaA*
	geNorm	*EF1b*	*GAPDH*	*RPS18*	*Actin*	*β-tubulin*	*VhaA*
	**Comprehensive**	***EF1b***	***GAPDH***	***RPS18***	***Actin***	***β-tubulin***	***VhaA***
Larvae (ds*IAP1*)	RF: Delta Ct	*VhaA*	*EF1b*	*β-tubulin*	*RPS18*	*Actin*	*GAPDH*
	RF: BestKeeper	*β-tubulin*	*EF1b*	*VhaA*	*GAPDH*	*RPS18*	*Actin*

	RF: Normfinder	*VhaA*	*EF1b*	*β-tubulin*	*RPS18*	*Actin*	*GAPDH*
	RF: geNorm	*VhaA*	*EF1b*	*β-tubulin*	*RPS18*	*Actin*	*GAPDH*
	**RF: Comprehensive**	***VhaA***	***EF1b***	***β-tubulin***	***RPS18***	***Actin***	***GAPDH***
	BestKeeper	*β-tubulin*	*EF1b*	*VhaA*	*GAPDH*	*RPS18*	*Actin*
	NormFinder	*VhaA*	*EF1b*	*β-tubulin*	*RPS18*	*Actin*	*GAPDH*
	geNorm	*VhaA*	*β-tubulin*	*EF1b*	*RPS18*	*GAPDH*	*Actin*
	**Comprehensive**	***VhaA***	***β-tubulin***	***EF1b***	***RPS18***	***GAPDH***	***Actin***
Life Stages	RF: Delta Ct	*Actin*	*VhaA*	*RPS18*	*EF1b*	*β-tubulin*	*GAPDH*
	RF: BestKeeper	*Actin*	*VhaA*	*EF1b*	*β-tubulin*	*RPS18*	*GAPDH*
	RF: Normfinder	*Actin*	*VhaA*	*EF1b*	*RPS18*	*β-tubulin*	*GAPDH*
	RF: geNorm	*RPS18*	*VhaA*	*Actin*	*β-tubulin*	*EF1b*	*GAPDH*
	**RF: Comprehensive**	***Actin***	***VhaA***	***RPS18***	***EF1b***	***β-tubulin***	***GAPDH***
	BestKeeper	*Actin*	*VhaA*	*EF1b*	*β-tubulin*	*RPS18*	*GAPDH*
	NormFinder	*Actin*	*VhaA*	*EF1b*	*RPS18*	*β-tubulin*	*GAPDH*
	geNorm	*VhaA*	*RPS18*	*Actin*	*β-tubulin*	*EF1b*	*GAPDH*
	**Comprehensive**	***Actin***	***VhaA***	***RPS18***	***EF1b***	***β-tubulin***	***GAPDH***
Female Tissues	RF: Delta Ct	*EF1b*	*GAPDH*	*Actin*	*VhaA*	*β-tubulin*	*RPS18*
	RF: BestKeeper	*VhaA*	*Actin*	*EF1b*	*GAPDH*	*β-tubulin*	*RPS18*
	RF: Normfinder	*GAPDH*	*EF1b*	*β-tubulin*	*Actin*	*VhaA*	*RPS18*
	RF: geNorm	*Actin*	*EF1b*	*VhaA*	*GAPDH*	*β-tubulin*	*RPS18*
	**RF: Comprehensive**	***EF1b***	***Actin***	***GAPDH***	***VhaA***	***β-tubulin***	***RPS18***
	BestKeeper	*VhaA*	*Actin*	*EF1b*	*GAPDH*	*β-tubulin*	*RPS18*
	NormFinder	*GAPDH*	*EF1b*	*Actin*	*β-tubulin*	*VhaA*	*RPS18*
	geNorm	*Actin*	*EF1b*	*VhaA*	*GAPDH*	*β-tubulin*	*RPS18*
	**Comprehensive**	***EF1b***	***Actin***	***GAPDH***	***VhaA***	***β-tubulin***	***RPS18***
VSV Injected	RF: Delta Ct	*EF1b*	*RPS18*	*Actin*	*VhaA*	*β-tubulin*	*GAPDH*
	RF: BestKeeper	*GAPDH*	*RPS18*	*EF1b*	*Actin*	*VhaA*	*β-tubulin*
	RF: Normfinder	*RPS18*	*EF1b*	*Actin*	*VhaA*	*β-tubulin*	*GAPDH*

	RF: geNorm	*Actin*	*RPS18*	*EF1b*	*VhaA*	*β-tubulin*	*GAPDH*
	**RF: Comprehensive**	***RPS18***	***EF1b***	***Actin***	***GAPDH***	***VhaA***	***β-tubulin***
	BestKeeper	*GAPDH*	*RPS18*	*EF1b*	*Actin*	*VhaA*	*β-tubulin*
	NormFinder	*EF1b*	*RPS18*	*Actin*	*VhaA*	*β-tubulin*	*GAPDH*
	geNorm	*Actin*	*RPS18*	*EF1b*	*VhaA*	*β-tubulin*	*GAPDH*
	**Comprehensive**	***EF1b***	***RPS18***	***Actin***	***GAPDH***	***VhaA***	***β-tubulin***
BTV Injected	RF: Delta Ct	*EF1b*	*Actin*	*RPS18*	*β-tubulin*	*GAPDH*	*VhaA*
	RF: BestKeeper	*Actin*	*RPS18*	*EF1b*	*β-tubulin*	*GAPDH*	*VhaA*
	RF: Normfinder	*EF1b*	*Actin*	*RPS18*	*β-tubulin*	*GAPDH*	*VhaA*
	RF: geNorm	*Actin*	*EF1b*	*β-tubulin*	*RPS18*	*GAPDH*	*VhaA*
	**RF: Comprehensive**	***EF1b***	***Actin***	***RPS18***	***β-tubulin***	***GAPDH***	***VhaA***
	BestKeeper	*Actin*	*RPS18*	*EF1b*	*β-tubulin*	*GAPDH*	*VhaA*
	NormFinder	*EF1b*	*Actin*	*RPS18*	*β-tubulin*	*GAPDH*	*VhaA*
	geNorm	*EF1b*	*Actin*	*β-tubulin*	*RPS18*	*GAPDH*	*VhaA*
	**Comprehensive**	***EF1b***	***Actin***	***RPS18***	***β-tubulin***	***GAPDH***	***VhaA***

[A]RefFinder (RF) reports rankings for each of the other analysis tools (BestKeeper, NormFinder, geNorm) and includes the delta Ct ranking when creating a comprehensive rank. Bolded text call attention to comprehensive ranks.

Supplementary Table S4. Pairwise V score values for each of the experiments in this study as determined by the geNorm analysis. A value of 0.15 or greater suggests a significant contribution of adding another reference gene. The experiments were cells treated with water or varying concentrations of ds*eGFP* (Cells (G)), cells treated with ds*eGFP* or varying concentrations of ds*VhaA* (Cells (V)), larvae treated with ds*eGFP* or ds*IAP1* (Larvae (I)), midge developmental stages (Stages), midge body parts or tissue groups (Body), and female midges injected with vesicular stomatitis virus (VSV) or bluetongue virus (BTV).

	**Cells (G)**	**Cells (V)**	**Larvae (I)**	**Stages**	**Body**	**VSV**	**BTV**
**V2/V3**	0.00213	0.00562	0.00482	0.01246	0.01660	0.00222	0.00273
**V3/V4**	0.00180	0.00487	0.00329	0.01410	0.01861	0.00304	0.00249
**V4/V5**	0.00132	0.00932	0.00328	0.01223	0.01615	0.00291	0.00376
**V5/V6**	0.00147	0.02785	0.00366	0.02411	0.02514	0.00489	0.00391

References for Supplementary Materials

1 Mills, M. K., Nayduch, D. & Michel, K. Inducing RNA interference in the arbovirus vector, *Culicoides sonorensis*. *Insect Mol. Biol.* **24**, 105-114 (2015). https://doi.org:10.1111/imb.12139

2 Yao, J., Rotenberg, D., Afsharifar, A., Barandoc-Alviar, K. & Whitfield, A. E. Development of RNAi methods for *Peregrinus maidis*, the corn planthopper. *PLoS One.* **8**, e70243 (2013). https://doi.org:10.1371/journal.pone.0070243
